# Wet Chemical-Synthesized Low-Loss Dielectric Composite Material Based on CuCl-Cu_7_S_4_ Nanoparticles and PVDF Copolymer

**DOI:** 10.3390/polym17131845

**Published:** 2025-06-30

**Authors:** Alexander A. Maltsev, Andrey A. Vodyashkin, Evgenia L. Buryanskaya, Olga Yu. Koval, Alexander V. Syuy, Sergei B. Bibikov, Irina E. Maltseva, Bogdan A. Parshin, Anastasia M. Stoynova, Pavel A. Mikhalev, Mstislav O. Makeev

**Affiliations:** 1 Department of Electronics of Organic Materials and Nanostructures, N.M. Emanuel Institute of Biochemical Physics (IBCP), Russian Academy of Science (RAS), 119334 Moscow, Russia; aam.0205@yandex.ru (A.A.M.); sbb.12@yandex.ru (S.B.B.); irine4593berd@mail.ru (I.E.M.); 2 Laboratory of Ferroelectric Polymers, Bauman Moscow State Technical University, 105005 Moscow, Russia; buryanskayael@bmstu.ru (E.L.B.); parshbgal@bmstu.ru (B.A.P.); pamikhalev@bmstu.ru (P.A.M.); m.makeev@bmstu.ru (M.O.M.); 3 Laboratory of Physics of Oxide Ferroelectrics, Department of Materials Science of Semiconductors and Dielectrics, National University of Science and Technology MISIS, 119049 Moscow, Russia; 4 National Research Center “Kurchatov Institute”, 123182 Moscow, Russia; o.yu.koval@gmail.com; 5 Department of General Physics, Perm National Research Polytechnic University, 614990 Perm, Russia; alsyuy@xpanceo.com; 6 Institute of Pharmacy and Biotechnology, RUDN University, 117198 Moscow, Russia; stoynova-am@rudn.ru

**Keywords:** polymer dielectric, composite, flexible electronic, low-loss dielectric, polyvinylidene fluoride, copper sulfide

## Abstract

Polymer composites with high dielectric permittivity (>10) and low dielectric loss are critical for energy storage and microelectronic applications. This study reports on a semi-transparent composite of a PVDF copolymer filled with Cu_7_S_4_ nanoparticles synthesized via a wet chemical route. Only a small content (6%) of copper sulfide increases the dielectric permittivity of the material from 10.4 to 15.9 (1 kHz), maintaining a low dielectric loss coefficient (less than 0.1). The incorporated nanoparticles affect the morphology of the composite film surface and crystalline phases in the whole volume, which was studied with FTIR spectroscopy, differential scanning calorimetry and scanning probe microscopy.

## 1. Introduction

Polymer composite materials with increased dielectric permittivity have many applications in various fields of engineering: capacitors, field-effect transistors and radio-absorbing materials [1,2,3]. Vinylidene fluoride (PVDF) copolymers are often used as binders in materials with increased dielectric permittivity because VDF copolymers and PVDF-based composites have high dielectric permittivity [4,5], transparency in the visible range and chemical resistance, and are suitable for making composite materials either from melts or organic solutions [6,7]. High dielectric permittivity and low intrinsic losses allow for the obtainment of both low-dielectric-loss materials [4,8,9,10] and materials with increased dielectric losses for use as radio-absorbing materials [11,12,13] on the basis of PVDF. Among the radar-absorbing materials with high dielectric losses are materials with additives of transition metal sulfides [12], for example, ZnS [14], CdS [14], MoS_2_ [15,16], CoS [17], Ni_x_S_y_ [13,18] and Cu_x_S_y_ [2,3,16]. Transition metal sulfides are often not stoichiometric compounds [13] or do not have a constant composition at all; in the synthesis of nanoparticles, the chemical composition of the product often depends on the initial ratio of reagents [19,20,21]. Thus, methods of selective synthesis can be used to obtain CuS (layered spherical nanoparticles [3], loose branched structures [22]), Cu_2_S (thin films [23], wine-like grapes [24], hexagonal prisms [25]), Cu_1_._8_S [26] and Cu_1_._75_S (chains of nanoparticles of nanostrings [19], hollow nanoparticles [27], snowflakes [28], hexagonal prisms [29]).

The electrophysical properties of copper sulfides with different chemical and phase compositions may differ significantly and strongly depend on the method of synthesis. For example, the electrical conductivity of semiconducting copper sulfides of Cu_1.8_S—Cu_2_S varies from 100 S/cm (plasma synthesis) to 3000 S/cm (wet chemical synthesis) [25]. Often in works devoted to composite materials, the electrophysical properties of bulk fillers cannot be measured, and data are given only for the prepared nanocomposite material.

The electrophysical properties of composite materials based on PVDF and copper sulfides have not been widely investigated yet. However, it is interesting that the addition of wet-synthesized CuS to PVDF increases both the real and imaginary parts of the dielectric permittivity of the materials by up to 20–30 (depending on the concentration of nanoparticles), but the same content of CuS nanoparticles in paraffin does not affect the dielectric parameters of the composite [3]. CuS nanoparticles in a shell of reduced graphite oxide [2] give the same effect when used in composites based on paraffin and PVDF. CuS nanoparticles grown by layer-by-layer precipitation [30] increase the dielectric permittivity of polyvinyl alcohol-based composites by up to 100 at low frequency. Thus, a significant increase in the dielectric permittivity of composites seems to occur in matrixes that chemically interact with copper ions (like polyvinyl alcohol) or matrixes that form hydrogen bonds with DMF-capped nanoparticles (like PVDF). An inert matrix, like paraffin, shows a very small increase in dielectric permittivity.

In this work, we present a semi-transparent composite dielectric material with increased dielectric permittivity based on non-stoichiometric copper sulfide (Cu_7_S_4_) incorporated in a vinylidene fluoride copolymer matrix. The partial transparency and increased dielectric permittivity of the material extend its applications to new domains of technology, e.g., dye-sensitized solar cells, sensor screens, chemical sensors, etc. [31].

Cu_7_S_4_ nanoparticles are synthesized from CuCl_2_∙4H_2_O and Na_2_S∙9H_2_O in mixed organic–water media (dimethylformamide (DMF) + propylene glycol + water from salt crystallohydrates). Both of the organic solvents used are able to weakly functionalize the particles’ surfaces and provide hydrogen bonding with PVDF copolymer chains without additional capping agents. Such functionalization of nanoparticles is compatible with PVDF and prevents their agglomeration and oxidation. Moreover, a colloidal solution of as-synthesized nanoparticles may be mixed with a polymer solution without polymer precipitation, which usually occurs in contact with water or alcohol solutions.

The choice of copper chloride as a precursor is based on its good solubility in DMF, as well as on the insignificant influence of residual CuCl_2_ on the properties of PVDF composites [32]. A side product of the reaction, CuCl, shows nonlinear electrophysical properties in dielectric composite materials based on PVDF [33].

## 2. Materials and Methods

### 2.1. Main Materials

A copolymer of vinylidene fluoride (94%) and tetrafluoroethylene 6% was purchased from Halopolymer, Russia. The electrophysical properties and crystalline phase content of the copolymer were investigated previously [34]. The solvents (isopropyl alcohol, acetone and dimethylformamide, all of 99% purity) were purchased from Component-Reactive, Russia. Ammonia solution (25%) and the metal salts (98% purity) Na_2_S∙9H_2_O and CuCl_2_∙4H_2_O were purchased from Russchem, Russia.

### 2.2. Material Preparation

**Figure 1:**
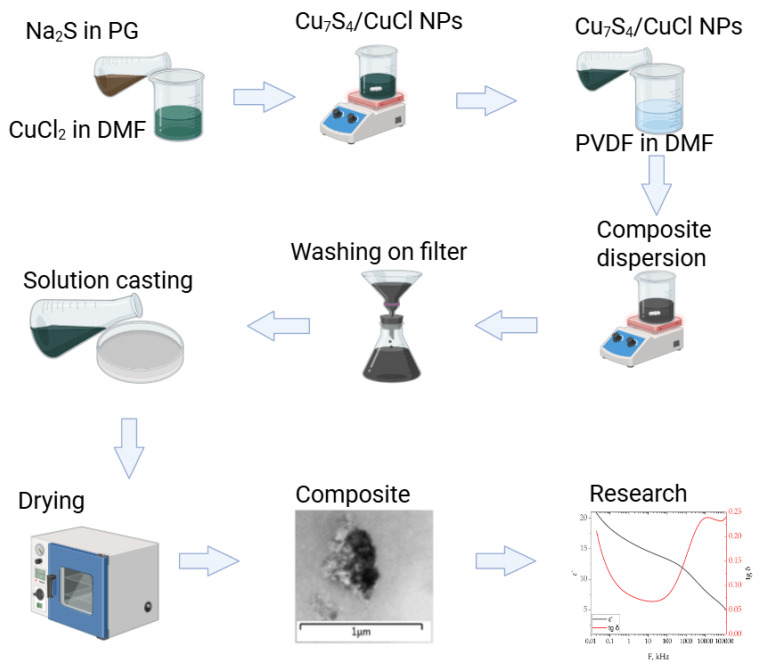
Scheme of composite synthesis process.

A total of 1.20 g (0.005 mol) of sodium sulfide 9-hydrate was ground in a mortar with 3.60 g of propylene glycol to a homogeneous white suspension and 1.04 g (0.005 mol) of copper (II) chloride 4-hydrate was dissolved in 3.12 g of DMF. The copper chloride solution was added dropwise to the sodium sulfide suspension with continuous grinding. Then, the yellow-brown suspension was poured into 36 g of DMF and stirred until homogeneous for 15 min using an overhead stirrer. A total of 4.5 g of PVDF powder was added to the resulting suspension and dissolved for 2 h at a temperature of 60 °C under the influence of ultrasound. The resulting suspension was slowly poured into a precipitation solution consisting of 100 g of distilled water, 24 g of isopropyl alcohol, and 1 g of ammonium hydrate. The vessel with the precipitation solution should be immersed in an ultrasonic bath. After precipitation, the vessel with the reprecipitated composite material was kept in an ultrasonic bath at a temperature of 60 °C for 30 min. The yellow-brown polymer sponge formed during precipitation was then crushed using scissors into fragments no larger than 5 × 5 × 10 mm and immersed for 30 min in an ultrasonic bath with a washing solution consisting of 90 g of water, 9.5 g of isopropyl alcohol, and 0.5 g of ammonia hydrate. The washing procedure was repeated three times until the blue color of the washing solution and the white coating on its surface disappeared, i.e., until unreacted copper salts and colloidal sulfur were removed from the system. After washing, the sponge was dried to a constant weight (approximately 5 g) at a temperature of 70 °C. The granules of the composite material changed color from yellow-brown to gray with small areas of dark olive color during the drying process. The dried composite material granules were dissolved at a temperature of 50 °C and the impact of ultrasound in a mixture consisting of 30% DMF and 70% acetone. The ratio of the mass of the granules to the solvent was 1:9. The solution was a cloudy olive-colored liquid. The solution was filtered through a syringe filter (PTFE), and the volume of the filtered portion was approximately 80% of the initial mass of the solution. The part that did not pass through the filter had a darker shade. The scheme of synthesis is given in Figure 1.

To compare the properties of solution-casted pure polymer film and composite film, a sample of pure polymer was dissolved in the same solvent mixture (30% DMF and 70% acetone) and the solution was dried on a Petri dish in the same conditions as the composite film. Three samples of pure polymer and two samples of composite film were prepared as described above.

### 2.3. XRD Study of Composite Material

The composition features of the studied system were assessed using the powder X-ray diffraction method (XRD). Structural and phase analysis of the investigated composites were carried out at room temperature on a Thermo ARL X’TRA powder diffractometer (Bruker, Germany) in the Bragg–Brentano geometry, with the radiation generated by an X-ray tube with Ni-filtered Cu Kα radiation (λ = 1.5418 Å, ≈8 keV). The used experimental conditions were 2θ = 22–82° with a step of 0.02°. The lattice cell parameters were evaluated using the Rietveld refinement [35] implemented in the FullProf (Winplotr) software package [36].

### 2.4. TEM Study of Composite Structure

Crystalline structure was studied with transmission electron microscopy (TEM) using a JEOL JEM–2100 field emission gun TEM (Tokyo, Japan) operating at 200 kV (point-to-point resolution of 0.19 nm in TEM mode). For TEM studies, the original polymer was dissolved in acetone and transferred to the carbon-coated TEM grid surface. The compositional analysis was performed with scanning transmission electron microscopy (STEM) (Tokyo, Japan) in energy-dispersive X-ray spectroscopy (EDX) mode [37]. Analysis of TEM images (particle size distribution) was performed with ImageJ 1.54g software.

### 2.5. Raman Spectroscopy

Raman spectra were collected with a PerkinElmer RamanStation 400F spectrometer (PerkinElmer, Hopkinton, MA, USA) with a laser wavelength of 785 nm. Laser power was set at 30 mW, with an acquisition time of 15 s and three scans.

### 2.6. FTIR Spectroscopy

ATR-FTIR spectra of the samples were obtained with an FTIR spectrometer FT-803 (Simex, Novosibirsk, Russia) with a diamond single-reflection ATR unit. The ATR-FTIR spectra were collected in the range 550–1800 cm^−1^ at a resolution of 2 cm^−1^.

FTIR absorbance spectra were obtained with a PerkinElmer 1760X (PerkinElmer, USA), at a range of 420–4000 cm^−1^ and resolution of 4 cm^−1^.

### 2.7. PVDF Phase Content Calculations

Phase content calculations were performed according to the method described in [38] with spectra deconvolution and subsequent curve fitting using Fityk software. Voigt peak shape and the Levenberg–Marquardt fitting algorithm were used. Total electroactive phase content (*β* and *γ* phases) was determined according to the following formula:

(1) $$F_{ea}=\frac{A_{840}}{1.26\cdot A_{760}+A_{840}}$$

where *A*_760_ and *A*_840_—amplitudes of α phase peak 760 cm^−1^ and (*β* + *γ*) peak 840 cm^−1^, respectively.

Pure *β* phase content was calculated with the formula:

(2) $$F_{\beta }=F_{ea}\cdot \frac{A_{1275}}{A_{1275}+A_{1233}}$$

where *A*_1275_ and *A*_1233_ are amplitudes of the *β* and *γ* phases, respectively.

### 2.8. Differential Scanning Calorimetry

The samples’ thermal properties and crystallinity degree were determined by differential scanning calorimetry (DSC) on a NETZSCH DSC 204F1 Phoenix device (NETZSCH-Gerätebau GmbH, Selb, Germany). The samples were placed for measurement in the aluminum crucibles, and they were heated in the temperature range of 25–200 °C at the heating rate of 2 K/min in the argon medium.

The DSC curves were applied to compute the film crystallinity degree using the simplified Formula (3):

(3) $$\chi_{c}=\frac{\Delta H_{m}}{\omega (a\cdot \Delta H_{m}^{\alpha }+b\cdot \Delta H_{m}^{\beta }+c\cdot \Delta H_{m}^{\gamma })},$$

where ∆H_m_ is the film melting enthalpy; $\Delta H_{m}^{\alpha }$ is the melting enthalpy of the α-phase, which is 93.07 J/g [39,40]; $\Delta H_{m}^{\beta }$ and $\Delta H_{m}^{\gamma }$ are the melting enthalpies of the β- and γ-phases, respectively, which are both equal to 103.4 J/g; *a*, *b* and *c* are the relative contents of the α-, β- and γ-phases, calculated from absorbance FTIR spectra; and *ω* is the content of PVDF in the whole material (100% for pure PVDF and 94% for the composite in assumption of its chemical composition described above).

### 2.9. Scanning Probe Microscopy of Composite Surface

The piezoelectric and morphological properties of the material were studied using an NTEGRA Prima atomic force microscope (NT-MDT SI, Zelenograd, Russia). Cantilevers with a platinum conductive coating FMG01/Pt (Tipsnano, Tallinn, Estonia) were used for the measurements. The measurements were carried out in semi-contact mode with a scanning speed of 0.5 Hz and a lifting height of approximately 50 nanometers. The experimental data were processed using the Gwyddion 2.67 software (Czech Metrology Institute, Czech Republic). The RMS roughness (R_q_) values of the films were calculated from scanning probe microscopy data using Equation (3). The instrument error in roughness measurement was approximately 50 picometers.

(4) $$R_{q}=\sqrt{\frac{1}{S}\int_{S}{\left[h\left(x,y\right)\right]}^{2}dxdy,}$$

where *S* is the surface area on which measurements are made and *h*(*x*,*y*) is the height of the surface at the point (*x*, *y*).

In the piezoelectric response force microscopy (PFM) mode, patterns of vertical (VPFM) and lateral piezoelectric response (LPFM) signal distribution were obtained, measurements were carried out at a fixed frequency of 120 kHz and a voltage of 5 V was applied to the cantilever. In the Kelvin probe microscopy (KPFM) mode, patterns of the distribution of surface potential signals were obtained and their histograms were constructed.

### 2.10. Dielectric Permittivity Measurements

Measurements of the real part of dielectric permittivity and dielectric loss coefficient were performed using a Wayne Kerr 6500P precision high-frequency RLC meter integrated into an automated measurement system. Copper electrodes in the form of discs with a diameter of 10 mm were applied to the opposite sides of the sample (film) under study by magnetron sputtering. The electrodes, in turn, were connected to the measuring terminals of the device by strips of the highly conductive fabric “Tainhou 230T black” or similar with a surface resistance of 10^−2^ Ohm and a width of 1 mm, fixed over the electrodes. A continuous spectrum of measured values was obtained with a rather dense set of frequencies (from 501 to 1001 points in the frequency range from 20 Hz to 120 MHz) set by the program installed on the control computer. After setting each frequency, the measurement of the real and imaginary components of impedance for a monochromatic signal in the representation of equivalent capacitance and resistance was carried out. After that, taking into account the geometry of the sample, the conversion to the components of the complex dielectric constant and dielectric loss coefficient was carried out using the following expressions (SI system of units):

(5) $$\varepsilon =C\cdot \frac{h}{S\cdot \varepsilon_{0}}$$

(6) $$\varepsilon \prime \prime =1.8\cdot 10^{10}\cdot \frac{h}{S\cdot R\cdot f}$$

(7) $$tg\delta =\frac{\varepsilon \prime \prime }{\varepsilon }$$

where *h* is the film thickness, *S* is the square of metal electrodes (deposited on opposite sides of the film), *C* is the measured capacity, *R* is the measured resistance, *f* is the frequency and ε_0_ is a dielectric constant.

### 2.11. Dielectrical Breakdown Measurement

The electrical strength was measured using a laboratory stand (a high-voltage current source), one of the poles of which was connected to an oil bath, and the other to an output electrode.

The samples were placed in a tub with petroleum jelly oil, and an overhead aluminum electrode with a diameter of 2.5 mm was placed on top of the sample. A polarizing voltage was applied to the sample from a high-voltage electrode.

The electrical strength of the material was measured at room temperature. The values of electrical strength were calculated using the two-parameter Weibull model, which describes the statistical distribution of a large number of electric field values using the following function:

(8) $$F\left(x\right)=1-\exp\left[-{\left(\frac{x}{\alpha }\right)}^{\beta_{b}}\right],$$

where *x* is the current value of the breakdown field, *α* is a certain characteristic field at which at least 63.2% of the tested samples are penetrated; and parameter *β* characterizes the variance of the breakdown field relative to the average value.

### 2.12. Optical Microscopy

Optical properties of the films were investigated with an A15.1302 Polarizing Microscope (Opto-Edu, Beijing, China).

### 2.13. UV-Vis Spectroscopy and Haze Measurement

Transmittance coefficients (T(λ)) of the film samples were measured in a range from 350 to 1000 nm using a two-beam spectrophotometer UV-3600i Plus (Shimadzu, Kyoto, Japan). To distinguish the absorbance and scattering effect of the films, two optical configurations were used for transmittance measurements: free space (*T_direct_*) with a slit width of 8 nm and an integrating sphere (*T_sphere_*) with a 32 nm slit width. In all the experiments, the spectral resolution of the slit was 5 nm. The total luminous transmittance (*τ_t_*) for the standard light source CIE D65 was calculated according to the method given in ISO 13468-2:2021 [41]. Haze, *H*(*λ*), was calculated with the Formula (8) according to the method described in ISO 14782:2021 [42]:

(9) $$H\;\left(\lambda \right)=\frac{T_{sphere}\left(\lambda \right)-T_{direct}\left(\lambda \right)}{T_{sphere}\left(\lambda \right)}$$

where *T_sphere_* (*λ*) is the transmittance coefficient, measured with an integrating sphere;

*T_direct_* (*λ*)—transmittance coefficient, measured in the free space configuration;
*λ* is a wavelength (nm).

## 3. Results and Discussion

### 3.1. Nanoparticle Characterization

#### 3.1.1. XRD Research

**Figure 2:**
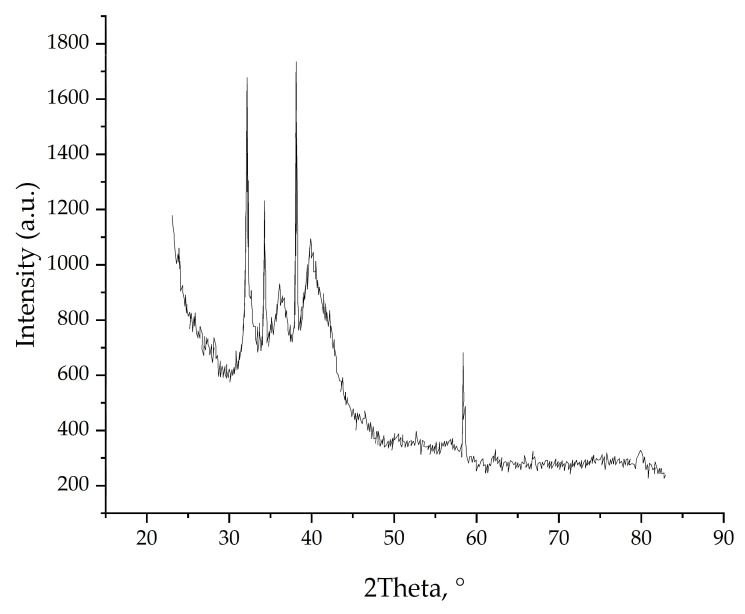
Powder X-ray diffraction pattern of synthesized composite film.

The composition of the crystallized particles was determined by obtaining powder X-ray diffraction patterns from fragments of film. The obtained powder X-ray diffraction patterns, shown in Figure 2, have a complex shape, the wide irregular signal can be attributed to the scattering signal of the polymer-host itself and the narrow reflections are described below.

In the X-ray diffraction pattern obtained from the light part of the film, bright crystalline reflections of only the phase roxbyite Cu_7_S_4_ [19] with a space group *P2_1_/c* studied in this work are marked with blue stars.

#### 3.1.2. TEM Research

TEM microphotographs at 500 nm scale show large amounts of almost spherical particles. The HRTEM microphotograph shows one selected particle with distinguishable interplanar distances which reflects the crystalline structure of nanoparticles.

**Figure 3:**
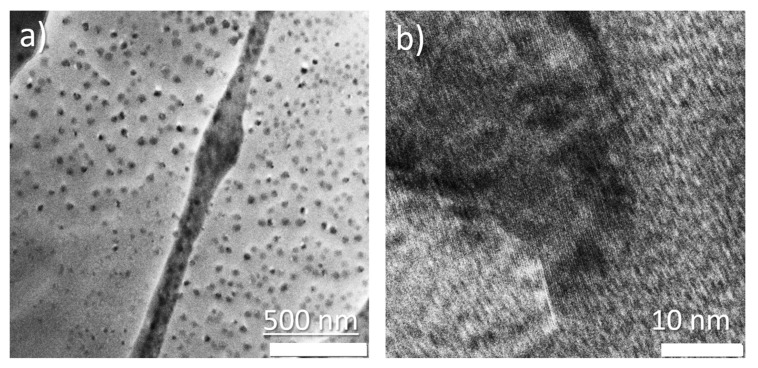
(**a**) TEM microphotographs of the synthesized nanoparticles, and (**b**) HRTEM image of the individual agglomerate. Interplanar distances may be seen.

**Figure 4:**
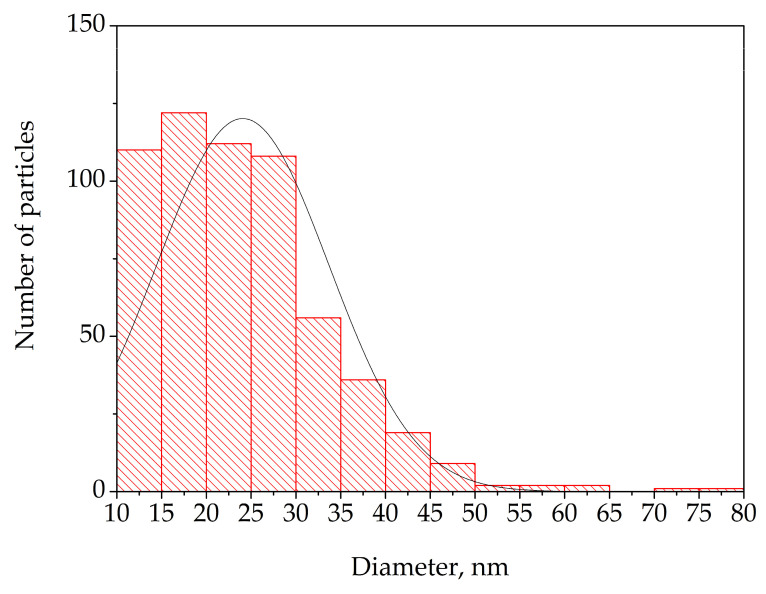
Particle distribution diagram.

According to the TEM image analysis (more than 500 particles from two TEM images with scale given in Figure 3a), the composite material contains nanoparticles of different sizes, predominantly from 10 to 40 nm. Figure 4 shows a diagram of particle distribution.

Some large aggregates with a size up to 350 nm with distinctive boundaries between particles also may be present. EDX analysis of such agglomerates shows almost equal content of chlorine and sulfur atoms. The TEM microphotographs of the synthesized nanoparticles are given in Figure 4.

#### 3.1.3. Chemical Composition

According to chemical structure investigations, the most possible phase of nanoparticles is roxbyite or anilite Cu_7_S_4_ (or 3Cu_2_S·CuS) with a small content of amorphous CuCl, showing the reduction of copper (II) to copper (I).

The possible presence of sodium thiosulphates and sulfites (as by-products of partially hydrolyzed and oxidized Na_2_S) leads to the reduction of copper (II) to copper (I), but Na_2_S_2_O_3_ and Na_2_SO_3_ have poor solubility in organic solvents and their residues cannot move through a filter.

So, the chemical reaction may be described with the following equation:

9CuCl_2_ + 8Na_2_S → 2CuCl + Cu_7_S_4_ + 4S + 16NaCl (10)

and the maximum content of copper sulfide in composite may not exceed 6.6% (mass) because of the solubility of CuCl in ammonia solutions used for composite washing.

### 3.2. Composite Chemistry Characterization

#### 3.2.1. Raman Spectroscopy

**Figure 5:**
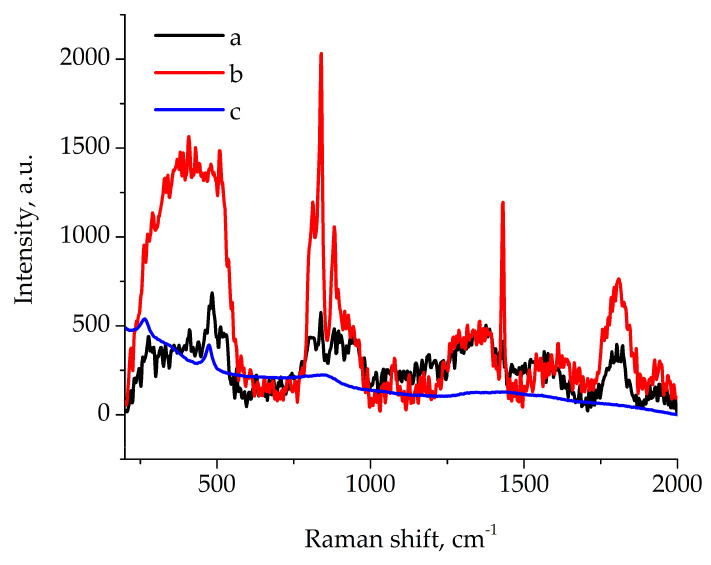
Raman spectra of composite film (a), pure film (b) and anilite mineral (Cu_7_S_4_) from database (c).

Raman spectra of pure polymer film and composite film were collected in the same conditions. Strong absorbance of composite near 800 nm (because of incorporated copper sulfides) restricts the use of high laser power or long acquisition time. The Raman spectrum of the composite was compared with the anilite (Cu_7_S_4_ mineral) spectrum from the RRUFF database [43] and showed similar bands (Figure 5).

Bands 812, 840, 880 and 1430 cm^−1^ are characteristic Raman bands for VDF copolymers. The most intensive band of 840 cm^−1^ with a weaker 812 cm^−1^ band predominantly show the β-phase of VDF copolymers in both pure polymer and composite film [44,45]. The weak bands at 260 and 475 cm^−1^ (Cu-S bond [46]) in the spectra of composite film shows the presence of Cu_7_S_4_.

#### 3.2.2. FTIR Characterization of VDF Copolymer Phases

The phase composition of the polymer matrix was studied using attenuated total reflection (ATR) IR spectroscopy. It was found that the α, β and γ phases were simultaneously present on the surface of the samples (peaks 760, 1276, 1234 cm^−1^) [37], which distinguishes them from extruded PVDF films, which contain very few γ phases [33]. To assess the distribution of nanoparticles in the composite layer, the ATR spectra were recorded from both sides of the film—the inner side (adjacent to the glass during casting) and the outer side (in contact with air).

**Figure 6:**
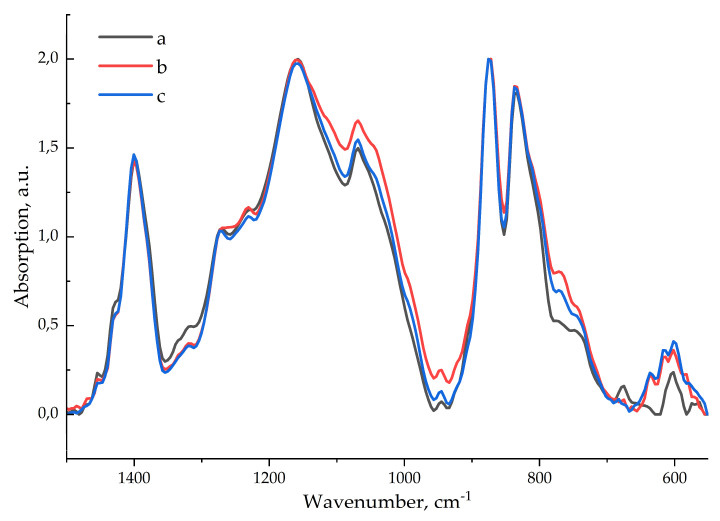
ATR-FTIR spectra of pure polymer (a), outer (b) and inner (c) sides of a composite film.

The IR spectra of the composite contain a weak band (which is not characteristic of pure PVDF) at 640 cm^−1^ (probably Cu-S), and increases in the intensity of the bands at 600 cm^−1^ (amorphous phase), 614 and 760 cm^−1^ (α-phase) are also observed. That is, the introduction of nanoparticles into the sample volume increases the content of the α-phase on the sample surface. The spectra of the outer and inner sides of the pure film coincide; therefore, only the spectrum of the inner side is given for comparison. ATR-FTIR spectra of the polymer and composite films are given in Figure 6.

The phase content of the polymer matrix (separately for the film volume and film surface) is given in Table 1.

Incorporated nanoparticles almost do not affect phase content on the film surface. However, in the whole composite layer, the β-phase content increases significantly because of the lowering content of the α- and γ-phases.

#### 3.2.3. DSC Analysis and Crystallinity Calculation

**Figure 7:**
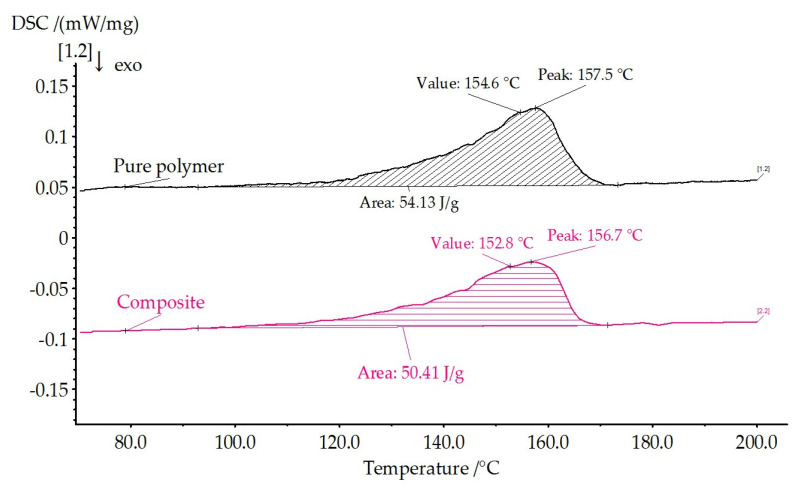
DSC curves of the first heating.

Figure 7 shows the curves of the first heats for the initial and composite films. Using Equation (1), the degree of crystallinity of the films is calculated, and the data are presented in Table 2.

Both samples are characterized by the presence of a pronounced peak in the beta phase. The shape of the curves of the first heating is similar. At the same time, the degree of crystallinity of the pure film is 52.9%, comparable with the degree of crystallinity of the composite film (52.1%).

Thermal parameters and crystallinity of the samples calculated from the FTIR and DSC measurements are given in Table 2.

The introduced nanoparticles do not strongly affect the crystallinity of the composite film, which correlates with FTIR data (almost the same relative intensity of the (β + γ)-phase peak 840 cm^−1^ and the mild difference in the 763 and 614 cm^−1^ characteristic peaks of the α-phase).

### 3.3. Composite Film Characterization

#### 3.3.1. Scanning Probe Microscopy

The morphological and piezoelectric properties of the pure and composite films were studied using scanning probe microscopy, and the data are presented in Table 3. The topography of the film surface was obtained from two sides. The side that was in contact with the air during crystallization in air was designated as the outer side, and the side that was in contact with the glass during crystallization in a Petri dish was the inner side.

**Figure 8:**
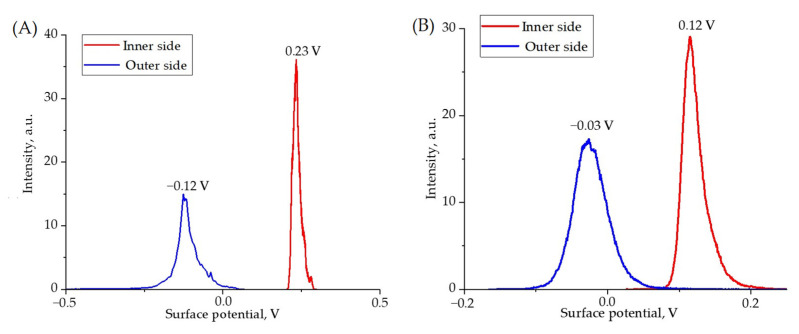
Surface potential distribution histograms for pure film (**A**) and composite film (**B**).

The surface potential signals obtained by Kelvin probe microscopy differ for different sides of the film (Figure 8), which indicates a difference in the electrophysical properties of the surface of the material. The difference in electrophysical properties may be due to different crystallization conditions.

The signs of the surface potential change in a similar way for both pure film and composite film. Such a distribution of surface potential signals may indicate the presence of spontaneous polarization in films. At the same time, the surface potential signals for the pure film are higher.

**Figure 9:**
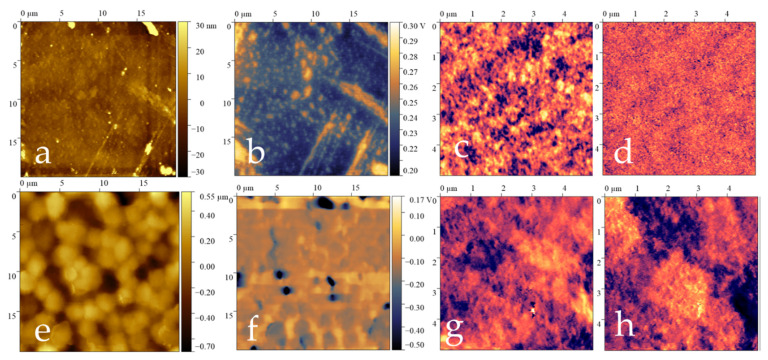
SPM data for pure film, where the top row is the inner side of the film, and the bottom row is the outer side of the film: (**a**,**e**)—topography, (**b**,**f**)—surface potential distribution maps, (**c**,**g**)—vertical piezoresponse signals, (**d**,**h**)—lateral piezoresponse signals.

**Figure 10:**
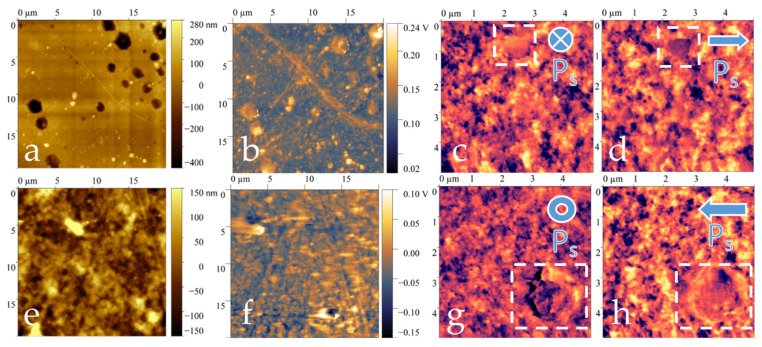
SPM data for composite film, where the top row is the inner side of the film, and the bottom row is the outer side of the film: (**a**,**e**)—topography, (**b**,**f**)—surface potential distribution maps, (**c**,**g**)—vertical piezoresponse signals, (**d**,**h**)—lateral piezoresponse signals.

Figure 9 and Figure 10 present the topographies, surface potential signal distributions and vertical and lateral piezoelectric response signals from both sides of the films.

The pure film has a more developed outer surface (RMS = 180 nm). Spherulites are present on the outside of the initial film (Figure 9e). Spherulites in polymers are spherical supramolecular structures formed in semi-crystalline polymers (such as PVDF, polyethylene and polypropylene) upon crystallization from a melt. They are radially symmetrical formations consisting of ordered crystalline lamellae radiating from a common center. The average diameter of the spherulites was 2.5 microns. For a composite film, no spherulites were observed on the surface. These data are consistent with the polarization microscopy data.

No spherulites were observed on the surface of the composite film. Spherical depressions with a height of 0.6 microns and a diameter of 2 microns can be seen on the inside of the composite film (Figure 10a). In addition, there are smaller protrusions measuring 0.3 microns in height and 1 micron in diameter on this side, which may be aggregates of nanoparticles. Due to these surface features, the inside of the film has a higher level of roughness, as measured by the root mean square (RMS) value, compared to the outside of the film. The RMS value for the inside of the film is 81 nanometers.

The composite film has a piezoelectric response. From the data of piezoelectric force microscopy, it was shown that both positively and negatively charged ferroelectric domains were present on the surface of the film. Figure 10c,d,g,h show the direction of the polarization vector in ferroelectric domains.

In this case, the macroscopic piezoelectric coefficients d_33_ for the composite film before and after polarization are 0; therefore, the material cannot be used in piezoelectric applications. But the polarized pure film has *d_33_* ~12 pC/N.

#### 3.3.2. Polarization Microscopy

Films of vinylidene fluoride copolymer with tetrafluoroethylene obtained by solvents are characterized by the fact that lamellar crystals form spherical regions of micron size in which the directions of polarizability along and across the radius differ. This causes the light passing through such a film, located between crossed polarizers, to experience depolarization. Calculations and experiments show that the two-dimensional scattering indicatrix in this case has the form of a 4-clover scattering pattern [47,48]. An optical indicatrix is a geometric model that visually represents the optical properties of anisotropic materials (crystals, polymers, liquid crystals) through the dependence of the refractive index on the direction of light propagation. For biaxial crystals, which include crystalline β-phase in PVDF copolymers, the optical indicatrix is a triaxial ellipsoid with three refractive indices, n_a_, n_β_ and n_γ_, where (n_a_ < n_β_ < n_γ_). According to the literature data [41], such structures are formed by crystallites of the paraelectric α-phase.

**Figure 11:**
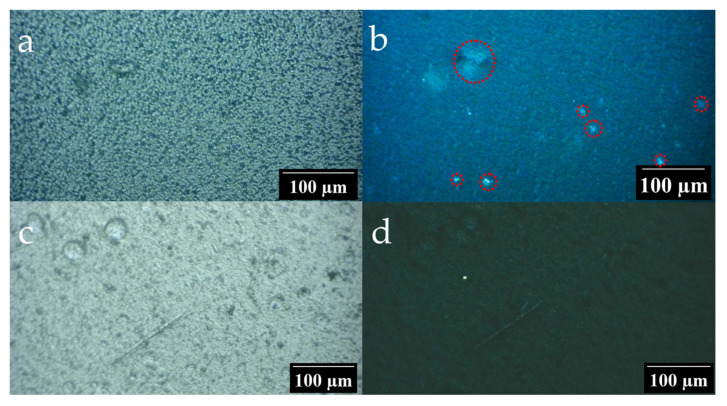
Polarization microscopy data: (**a**,**b**) are micrographs of the original film at 0° and 90° angles between the polarizer and analyzer, respectively. (**c**,**d**) are micrographs of the composite film at 0° and 90°.

The optical properties of composite films have been studied using polarization microscopy. Figure 11 shows micrographs of films at an angle between the polarizer and the analyzer of 0° (Figure 11a,c) and 90° (Figure 11b,d).

For a pure VDF/TFE copolymer film, weak birefringence was observed when the sample was placed between crossed polarizers and analyzers (Figure 11b). This indicates the presence of imperfect spherulites in the film.

There is no optical indicatrix for the composite film under consideration (Figure 11d). The film is practically optically isotropic. Slight scattering is observed only on surface defects. This may be because nanoparticles introduced into the polymer matrix prevent the formation of spherulites. On the one hand, surcharges can create spatial constraints for the rearrangement of macromolecules, making it difficult for the radial growth of spherulites. On the other hand, nanoparticles can act as additional nucleation centers, increasing the total number of nuclei. During this process, instead of large (~1–10 microns) spherulite structures, many small crystallites are formed. The formation of a large number of small crystallites helps to increase the transparency of the film.

#### 3.3.3. Optical Measurement

**Figure 12:**
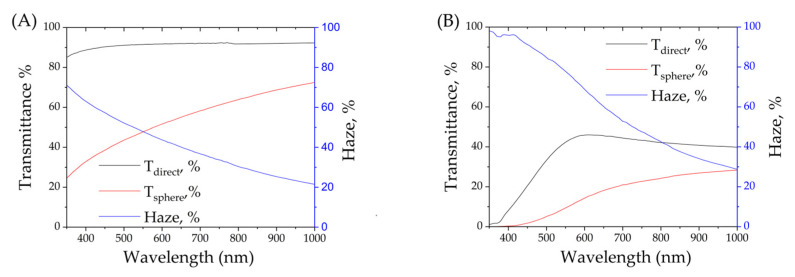
Transmittance with integrating sphere, direct transmittance and haze of 15 μm thick film of pure polymer (**A**) and composite (**B**).

To compare optical properties of the pure polymer and composite film, optical transmittance spectra were collected in two modes: direct transmittance and transmittance using an integrating sphere. Using two modes allowed us to calculate haze dependence on wavelength in optical range. Optical transmittance spectra and haze calculations are given in Figure 12.

The composite material films have low transmittance below 400 nm, which is typical for semiconductive nanoparticles of CuCl, especially n-doped [49,50]. The main absorbance (or reflectance?) peak of Cu_x_S_y_ also may be found in the range 350–375 nm [51]. The composite material shows maximum transparency in the range 550–650 nm, as with other materials based on Cu_7_S_4_ [51]. Partial transparency combined with scattering or absorbance in UV and near-UV bands may be used in sensors, dye-sensitized solar cells, UV-protecting coatings, etc.

### 3.4. Composite Electrical Properties Characterization

#### 3.4.1. Impedance Spectroscopy Measurement

**Figure 13:**
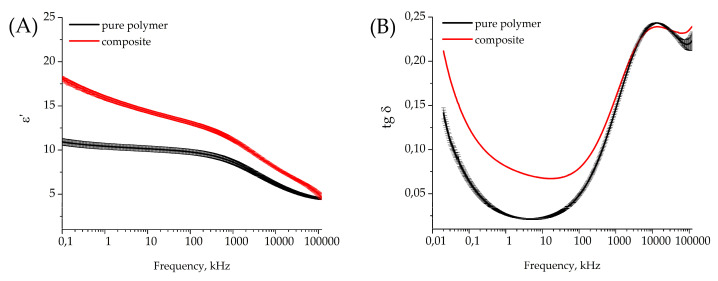
Frequency dependence of dielectric permittivity of the pure polymer and composite film (**A**) and dielectric loss coefficient (**B**) for pure polymer and composite film.

Dielectric parameters were measured for three samples of pure polymer film and two samples of composite film. All tested samples were covered with thermal sputtered copper electrodes (150 nm thickness, round shape of 10 mm diameter). One of two composite samples had a small defect after electrode sputtering, so the dielectric loss tangent was correctly calculated for only one composite sample because of electric leakage in the other sample. Dielectric permittivity (ε) and the dielectric loss coefficient (tg δ) for pure polymer and composite film are given in Figure 13A and Figure 13B, respectively.

The measurements show that embedded Cu_7_S_4_ nanoparticles in the PVDF copolymer increase the dielectric permittivity of the material at 1 kHz (10.4 ± 0.4 for pure polymer and 15.9 ± 0.2 for composite), maintaining a relatively low dielectric loss coefficient (0.026 ± 0.001 for polymer and 0.08 for composite).

#### 3.4.2. Electric Breakdown Field Strength Measurement

**Figure 14:**
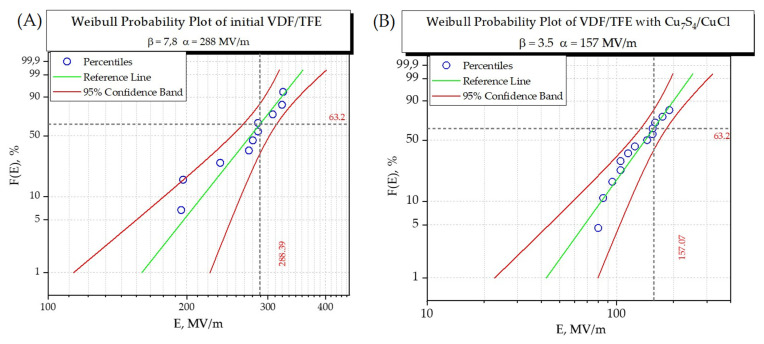
The magnitude of the electric breakdown field strength calculated using the Weibull function, where (**A**) is the initial film VDF-TFE and (**B**) is the composite film.

For a more complete characterization of the material as a low-loss dielectric, the electric strength of the material was measured (Figure 14).

It follows from the data presented in Figure 14 that the addition of nanoparticles reduces the electric strength of the material. This decrease in electric strength could be due to the disruption of the uniformity of the material, as well as the creation of additional breakdown pathways when nanoparticles are incorporated into the polymer matrix.

## 4. Discussion

The comparison of dielectric materials presented in this work and in other similar works is given in Table 4. Some works give data for low frequency (under 1 MHz, measured in impedance spectroscopy mode for thin films); other works give data for ultra-high frequency (over 1 GHz, measured in coaxial wire mode for thick materials).

The composite material presented in this work has dielectric parameters comparable with some other composite materials, for example, ZnO-PVDF nanocomposite. So, materials with graphite oxide and reduced graphite oxide show much higher dielectric permittivity at ultra-high frequency, but seem to be expensive due to using graphite oxide. Bismuth telluride is ferroelectric and its dielectric permittivity is high in most composites, but its fabrication requires high temperature and inert atmosphere. Nanoparticles of stoichiometric copper sulfide (CuS) mentioned in different works [2,3,30,55] provide high conductivity in different polymer matrices (high dielectric constant and high dielectric loss tangent simultaneously). The composite film mentioned in this work, like other composites filled with nanoparticles, has less electric strength than a pure polymer film because of the conductive nature of the nanofillers.

The combination of increased dielectric permittivity with relatively low dielectric losses and zero piezoeffect makes the material ideal for use as a dielectric for low-frequency film capacitors (e.g., for audio). The electric strength of the composite material is still sufficient to create capacitors based on it. According to the measurements, the electric breakdown strength E_b_ was found to be 157 MV/m, which is sufficient for using in flexible electronics [56]. This corresponds to a maximum operating voltage for a capacitor based on this material of 1.5 kV, with a dielectric thickness of 10 μm under the experimental conditions described in this study. Operating voltage up to 1.5 kV is sufficient for fixed capacitors for many general purposes [57].

## 5. Conclusions

This study demonstrates a scalable approach to fabricating low-loss dielectric films with ε ≈ 16 and tan δ ≈ 0.08 at 1 kHz. These properties, along with moderate transparency and breakdown strength (~157 MV/m), position the composite as a promising material for next-generation capacitors and optoelectronic components (sensor displays, protecting layers for solar cells, etc.). Incorporating Cu_7_S_4_ nanoparticles in a PVDF copolymer matrix significantly affects the surface morphology of the film, such as crystalline phase contents.

## Figures and Tables

**Table 1:** Phase content of polymer matrix.

Layer	α-Phase Content, %	β-Phase Content, %	γ-Phase Content, %
Pure polymer, both surfaces	32	32	36
Composite, inner side	36	29	35
Composite, outer side	35	27	38
Pure polymer, volume	10	43	47
Composite, volume	5	55	40

**Table 2:** Polymer matrix crystallinity calculation based on FTIR and DSC data.

Material	Measured Melting Enthalpy, J/K	Melting Temperature, °C		Phase Content, %		Crystallinity, %
		β	α	β + γ	α	
Pure polymer	54.13	154.6	157.5	90	10	52.9
Composite (94% polymer)	50.41	152.8	156.7	95	5	52.1

**Table 3:** Scanning probe microscopy data.

Sample	Side	RMS, nm	Surface Potential φ, V
Pure polymer	Inner side	9	0.23
	Outer side	180	−0.12
Composite polymer	Inner side	81	0.12
	Outer side	55	−0.03

**Table 4:** Comparison of electric properties of different PVDF-based dielectric materials.

Material	Frequency	Dielectric Constant, ε’	Dielectric Loss Tangent, tg δ	Electric Strength, MV/m	Reference
10% reduced graphite oxide/CuS in PVDF	2 GHz	24	~ 0.8	n/d	[2]
10% CuS in PVDF	2 GHz	12	0.05	n/d	[3]
Pure PVDF	2 GHz	~3	<0.15	n/d	[3]
Polypropylene	1 kHz	2.6	n/d	550	[52]
Polypropylene (isotactic)	1 kHz	2.2	0.0001	n/d	[53]
PolyK PVDF (pure)	1 kHz	11.5	<0.015	311	[4]
5% Cu@Graphite Oxide in PVDF	1 kHz	45	<0.1	n/d	[9]
10% Ni_x_S_y_ in PVDF	2 GHz	5	<0.15	n/d	[13]
10% ZnO in PVDF	1 kHz	10	0.026	95	[31]
10% Bi_2_Te_3_ in PVDF	1 kHz	385	0.20	~ 50	[54]
10% CuS in PVA/PVP blend	1 kHz	8000	~2.5	n/d	[55]
Cu_7_S_4_/CuCl (6% Cu) in VDF-TFE copolymer	1 kHz	15.9	0.08	157	This work
VDF-TFE copolymer	1 kHz	10.4	0.026	288	This work

## Data Availability

Dataset available on request from the corresponding author.

## Abbreviations

The following abbreviations are used in this manuscript:

PVDF	Poly(vinylidene fluoride)
DMF	Dimethyl formamide
PVA/PVP	Mixture of two polymers: polyvinyl alcohol and polyvinylpyrrolidone
n/d	No data
